# Orientations to Happiness between the Dark Triad Traits and Subjective Well-Being

**DOI:** 10.3390/bs10050090

**Published:** 2020-05-12

**Authors:** Pierpaolo Limone, Maria Sinatra, Lucia Monacis

**Affiliations:** 1 Department of Humanities, University of Foggia, 71100 Foggia, Italy; pierpaolo.limone@unifg.it (P.L.); lucia.monacis@unifg.it (L.M.); 2 Department of Educational Sciences, Psychology, Communication, University of Bari, 70121 Bari, Italy

**Keywords:** Narcissism, psychopathy, Machiavellianism, orientations to happiness, subjective well-being

## Abstract

Previous research investigated the linkage between the Dark Triad traits and subjective well-being, but the factors explaining individual differences in terms of cognitive strategies for achieving happiness remained poorly understood. This study (N = 460) examined the indirect effects of orientations to happiness in the link between dark personality traits and subjective well-being in terms of life satisfaction and positive emotion. Participants completed a questionnaire comprising the Dark Triad Questionnaire, the Orientations to Happiness scale, the Satisfaction with Life scale, and the PANAS. Descriptive statistics, bivariate and partial correlations, and structural equation model were applied to the data. Zero-order and partial correlations showed no significant associations of Machiavellianism and psychopathy with subjective well-being measures, and positive associations of narcissism with the three orientations to happiness and the two dimensions of subjective well-being. Indirect effects indicated that the bright side of narcissism sought the pursuit of the emotional component of SWB by adopting engaging activities. Further studies should replicate our findings.

## 1. Introduction

In recent years, the interest in well-being within the psychological field has grown rapidly in order to investigate the sources of happiness and the many aspects of human flourishing [1,2]. In this vein, research on well-being has been derived from two perspectives. The first, the hedonic approach, focuses on pleasure and happiness as well as on the achievement of well-being through the satisfaction of one’s desires. The second, the eudaimonic approach, implies the psychological well-being (PWB) [3] obtained by fulfilling one’s potential in the pursuit of meaningful goals. As for the hedonic perspective, it refers to the study of subjective well-being (SWB) operationalized in three components: more positive affect, less negative affect, and life satisfaction [4,5]. The first two components represent the more emotional and affective aspects of happiness, whereas the third is rather cognitively oriented, being based on the evaluations of one’s life circumstances.

Traditionally, hedonic and eudaimonic approaches were developed by the authentic happiness theory elaborated by Seligman [6], who added a third route to happiness, i.e., the pursuit of engagement, being—for him—all the three pathways important to live the ‘full life’. In line with this assumption, Peterson and colleagues [7] operationalized three distinct pathways or cognitive strategies to seek happiness: life of pleasure, which, hedonistically oriented, maximizes positive experiences giving importance to sensory pleasure for the attainment of a good life; life of engagement, which concerns highly engaging activities, thus producing a state of flow characterized by feelings of euphoria and a perception that time passed quickly [8]; and life of meaning, which, eudaimonically oriented, refers to activities that contribute to the greater general good, such as parenting, developing friendships or community services. Such a combined framework encouraged a flurry of research on individual differences in subjective well-being by taking into account the contribution of each of the three orientations [2,9,10].

While there are sufficient studies reporting the relationships of these orientations to happiness with SWB and personality traits based on the Five Factor Model [2,7,9], research examining linkages between orientations to happiness and dark traits is non-existent. Therefore, the current research intended to fill this gap by delineating individual differences in pursuing the subjective well-being by considering the Dark Triad Traits (DTT) as a general underlying personality construct to structure this information. Assumed as a valid candidate for the analyses of the socially malevolent personality domain, the Dark Triad personality cluster is a constellation of three conceptually distinct, but empirically overlapping traits: subclinical narcissism, Machiavellianism, and subclinical psychopathy [11]. Narcissism is typified by a strong sense of superiority, dominance and entitlement and by a grandiose sense of self [12]; Machiavellianism is characterized by glib social charm, tendencies toward strategic manipulation, and a lack of conventional morality [13]; and psychopathy refers to callous social attitudes, impulsivity, and interpersonal antagonism [14].

Within the dark traits research, prior studies investigated their associations with SWB [15,16]. However, the attempts to elucidate if and to what extent these personality dispositions might determine happiness and SWB have been somewhat equivocal or limited to a series of associations. For instance, Aghahabaei and colleagues’ study [15] reported no association between happiness, operationalized as global subjective happiness [17] and the three dark traits, measured by the Dirty Dozen scale. The same Aghahabaei with another colleague [18] showed consistent and positive relations between two measures of SWB, i.e., happiness in terms of the above-mentioned global subjective happiness, and life satisfaction evaluated by the 5-item Satisfaction with Life Scale [19], and narcissism, measured by the Short Dark Triad Scale [20]. In the same research, results showed negative correlations of Machiavellianism and psychopathy with SWB measures, although no significant predictive power of these traits emerged. Egan et al. [16] reported null relation of narcissism with SWB measures, evaluated by the Satisfaction with Life Scale and the Oxford Happiness Questionnaire, and negative associations between the remaining two traits with SWB measures. 

Based on these premises, we aimed at (1) exploring the links between DTT and SWB in order to provide further empirical evidence to the existing literature on the topic, and (2) extending previous knowledge of these associations by testing, through mediational analyses, the hypothesis of the instrumental nature of the DTT, according to which personality traits indirectly influence SWB. The instrumental theory assumes that certain situations or life experiences chosen by individuals influence their SWB. Within this indirect relationship, it might be argued that the existence of some cognitive mechanisms function as potential mediating factors capable of better clarifying the connections between DTT and SWB. 

Although the DTT entails a socially malevolent character with behavior tendencies toward self-promotion, emotional coldness, interpersonal manipulation, and aggressiveness, they manifest unique behavior. According to Jones and Paulhus’ conceptualization [20], DTT includes the grandiose variant of narcissism characterized by an ego-reinforcement. Hence, individuals high on this trait are generally known for holding very positive self-views [21,22,23,24,25], by adopting self-presentational strategies to garner positive feedback from others, as suggested by the dynamic self-regulatory process model [26]. In this sense, narcissists have been characterized as “social oriented” people [27] and, as they activate strategies for assertive self-enhancement [28], it would be likely to hypothesize that these individuals use a pleasure-oriented cognitive strategy that blunts the impact of life’s adversities, thus contributing to experiencing more frequent positive emotions. Consequently, we expected to find positive association between narcissism and the hedonic pathway, which, in turn, influences the positive affective component of SWB (H1a). 

Following two theoretical perspectives, i.e., the agency model [29] and the admiration vs. rivalry model [28], narcissism differs from the two “darker” personalities (Machiavellianism and psychopathy) because of its “brighter” elements or agentic features that characterize its assertive behaviors. The positive qualities lead these individuals to be more attractive and, to this purpose, to be actively engaged in ego-promoting. We supposed, therefore, positive links of narcissism with engagement orientation, which might positively influence both affective and cognitive aspects of SWB (H1b). 

The agentic features were further delineated by the agency-communion model [30] that shed light into two specific facets. First, individuals can be concentrated on their core self-motives of grandiosity, self-esteem, power, and entitlement *via agentic ways*, for example by overstating their seeming virtues on the agentic domain. Second, individuals can satisfy their core self-motives *via communal ways*, such as by exaggerating their apparent virtues on the communal domain. Following this distinction, [31] positive associations between these profiles and the affective and cognitive aspects of SWB were reported. As for the communal aspect, it was found to be related to a pro-social attitude combined with intrapersonal and interpersonal harmony. Hence, the bright side of narcissism that does not fit the definition of a “dark personality” trait [32]. 

Based on these premises, we supposed a tendency of narcissists to adopt the path of life of meaning in order to seek a personal harmony that could influence higher levels in both components of SWB (H1c). This relation was also hypothesized, given the already observed positive connection of extraversion with narcissism [11] and the indirect links of extraversion with life satisfaction via life of meaning [2].

With regard to the other two traits, given the explorative nature of our study, we expected similar patterns of associations when they were related to SWB measures and some differences when related to the three paths. Since psychopathy is characterized by deficits in affect (i.e., callousness) and by thrill seeking, individuals high on this trait manifest reckless antisocial behavior [33,34] and a dysfunctional impulsivity [35] that leads them to adopt short-term and more emotional-oriented strategies and to be engaged in activities in order to reach an immediate gain, thus compromising long-term interests. Consequently, we supposed negative associations of psychopathy with SWB measures and positive associations with negative affect, in line with previous findings [15,16] (H2a), and positive associations with the more hedonically oriented path, i.e., life of pleasure, and with life of engagement, (H2b), and negative associations with the life of meaning (H2c). The negative association of psychopathy with the more eudaimonic pathway was justified by the interpersonal antagonism linked to a wide array of socially undesirable outcomes [36].

Finally, as Machs high on this trait are characterized by cold selfishness, are less impulsive and tend to pay much attention to positive reputation by building alliances, we expected a positive linkage of Machiavellianism with pleasure orientation (H3a), no associations with both components of SWB measures, a positive association with negative affect (H3b), and no links with the path of meaning and engagement (H3c).

## 2. Materials and Methods

### 2.1. Participants and Procedure

A sample of 460 undergraduate Italian young adults (Men = 273, Women = 187; M = 22.17, SD = 4.15, Range = 18–56) voluntarily completed a paper-and-pencil survey during the psychological course. Participants were informed of the nature of the study, provided signed consent, completed a series of self-report measures, and were thanked and debriefed upon completion. The study was conducted in accordance with the Helsinki declaration and with the ethical rules of the Italian Psychological Association and it was approved by the ethics committee at the local University.

### 2.2. Measures

To measure the Dark Triad traits, we used an authors’ translated version of the Short Dark Triad [20] by using the back-translation procedure and in agreement with the widely accepted guidelines for the translation of measures in cross-cultural research. The measure is composed of 27 items (nine for each dimension), where respondents rate their agreement (1 = strongly disagree; 5 = strongly agree) with statements such as “I tend to manipulate others to get my way” (i.e., Machiavellianism), “I tend to lack remorse” (i.e., psychopathy), and “I tend to want others to admire me” (i.e., narcissism).

A first CFA was performed to evaluate its structural properties. Data showed poor fit indices, χ^2^ 635.216, df = 291, *p* ≥ 0.001; CFI = 0.80; RMSEA = 0.051, 90% CI = 0.045–0.056; SRMR = 0.084. Then a second CFA was performed using item parceling according to the item-total correlations assignment method [37]. Fit indices showed adequate values, χ^2^ 32.818, df = 10, *p* ≥ 0.001; CFI = 0.971; RMSEA = 0.071, 90% CI = 0.056–0.082; SRMR = 0.030. All factor loadings were significant and ranged from 0.496 to 0.772. Items were averaged to create a fairly internally consistent measure of individual differences in Machiavellianism (Cronbach’s α = 0.72), psychopathy (Cronbach’s α = 0.71), and narcissism (Cronbach’s α = 0.65). However, two items were excluded (for psychopathy, “I have never gotten into trouble with the law”, and for narcissism, “I insist on getting the respect I deserve”). This modification was also in line with an Italian study [38]. 

We used an authors’ translated version of the Orientations to Happiness Scale [7] following the above-mentioned guidelines for the translation of measures. Designed to measure three approaches to happiness, i.e., engagement (item example: “I seek out situations that challenge my skills and abilities”), meaning (item example: “I have a responsibility to make the world a better place”), and pleasure (item example: “Life is too short to postpone the pleasures it can provide), the scale comprises 18 items (six for each dimension) ranged on a 5-point Likert scale (1 = not at all; 5 very much). Alpha values were 0.63 (pleasure), 0.63 (engagement), and 0.65 (meaning). Given the lack of an Italian validation of the instrument, a CFA was run to evaluate the structural properties. Fit indices showed poor values, χ^2^ 357.849, df = 132, *p* ≥ 0.001; CFI = 0.734; RMSEA = 0.061, 90% CI = 0.054–0.069; SRMR = 0.063. Fit indices from the second CFA with parcels showed good values, χ^2^ 12.977, df = 6, *p* ≥ 0.001; CFI = 0.978; RMSEA = 0.050, 90% CI = 0.008–0.088; SRMR = 0.027. All factor loadings were significant and ranged from 0.484 to 0.806.

We measured life satisfaction with the 5-item Satisfaction With Life Scale (SWLS) [39]. The scale, ranged on a 7-point Likert scale, includes five items assessing global cognitive judgments of one’s life satisfaction (item example: “I am satisfied with my life”). Alpha value was 0.84.

Finally, we used the 20-item Positive/Negative Affect Schedule (PANAS) [40]. The scale consists of 10 adjectives, 10 referring to positive affect (i.e., enthusiastic, interested) and 10 negative affect (i.e., afraid, distressed). Respondents were asked to rate the extent to which they had experienced each feeling over the past few weeks using a 5-point Likert scale (1 = very slightly; 5 = extremely). Alpha values were 0.82 and 0.83, respectively.

### 2.3. Analysis Strategy

Correlations were performed to assess the associations among the variables of interest zero-order. Moreover, according to Furham and colleagues [41], partial correlations were also reported to factor out the influence of the other two traits in the patterns of associations with SWB measures and with three routes of happiness. In this way, we found out the independent contribution of each trait to the outcomes’ variables. The hypothesized instrumental nature of dark personality traits influencing SWB constructs via the three orientations to happiness was tested by using a structural equation model with latent variables. The exogenous and mediating variables were measured using parcels, satisfaction with life using its items as indicators, and positive affect using parcels. The structural equation model was evaluated using maximum likelihood (ML) as estimator. The significance of indirect effects was evaluated employing bootstrapping procedures (10,000 resamples) and the 95% bias-corrected confidence interval. The following indices were considered to assess the adequacy of the model: Chi square value (χ^2^) with the number of degrees of freedom, root mean square error of approximation (RMSEA), comparative fit index (CFI), and standardized root mean square residual (SRMR). The adequacy of a model is supported by a non-significant χ2, RMSEA values less than 0.06 (0.06 to 0.08, for a reasonable fit), CFI values close to 0.95 (0.90 to 0.95, for a reasonable fit), and SRMR less than 0.08 [42].

## 3. Results

The results from both correlational analyses shown in Table 1 confirmed: for narcissism, its positive associations with the three orientations to happiness and SWB, and its negative association with negative affect; for Machiavellianism and psychopathy, no associations with life satisfaction, positive associations with negative affect, life of pleasure and engagement. However, the positive linkage between engagement and Machiavellianism became not significant when holding constant the other two traits.

With regard to meaning, the findings confirmed no association with Machiavellianism and a weak negative association with psychopathy only in partial correlation, i.e., when controlling for the effects of the other two dark traits. Consequently, given that Machiavellianism and psychopathy were unrelated to positive affect and life satisfaction, the instrumental hypothesis of the indirect effect of personality on SWB constructs via the three paths of happiness was tested only for the trait of narcissism.

**Figure 1:**
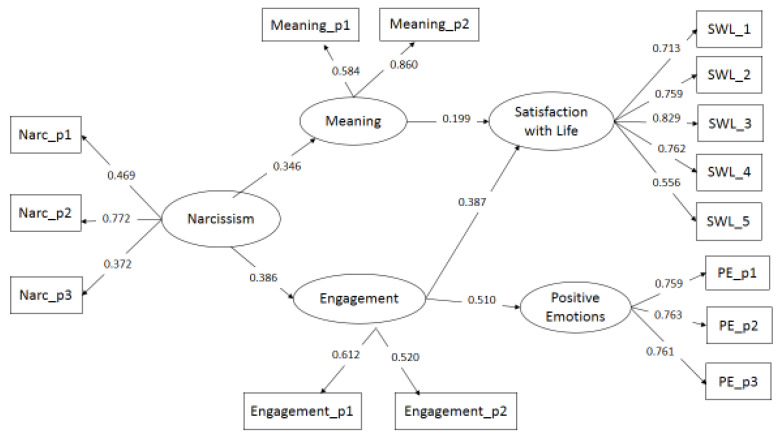
Mediation effects of paths of happiness in the relationship between narcissism and life satisfaction and positive emotion (N = 460). Only significant regression coefficients with standardized coefficients are reported.

The fit values of the first model were adequate, χ^2^(107) = 309.939, *p* < 0.001; RMSEA = 0.06, 95%CI: 0.05–0.07; CFI = 0.91; SRMR = 0.05. The standardized results partially supported our hypotheses. As predicted, life of meaning and engagement were positively related to narcissism and to the two criterion variables in the expected directions, whereas life of pleasure was positively connected to narcissism but unrelated with SWB measures. Consequently, a second model was evaluated by removing the pleasure orientation. The new index values showed better model fit, χ^2^(81) = 210.120, *p* < 0.001; RMSEA = 0.05, 95% CI: 0.04–0.06; CFI = 0.94; SRMR = 0.05. The relationships of narcissism with the cognitive and emotional aspects of well-being were mediated by the two routes (Figure 1). To test the significance of these indirect effects, bootstrap was applied, and the 95% bias-corrected confidence interval was used. Although all the indirect paths displayed in Figure 1 were significant, the reliable path was only from narcissism to positive emotion via life of engagement (Table 2), since the 95% confidence interval included zero for the other two paths, i.e., narcissism → meaning→ life satisfaction, and narcissism → engagement → life satisfaction.

## 4. Discussion

In light of the increasing attention on whether and to what extent dark personality traits seek happiness and subjective well-being, the present study intended to provide a more comprehensive view on this question by (1) examining the linkages between Dark Triad traits and SWB, and (2) testing the hypothesis of the instrumental nature of the dark personality traits. To this purpose, the mediating role of the three orientations to happiness, considered as cognitive mechanisms, was examined within these relationships. 

In general, our results were mixed and only partially supported the hypotheses. First, partial correlations showed no associations of Machiavellianism and psychopathy with SWB measures, although positive relations emerged between the two dark traits and negative affect. Consequently, our hypotheses (H2a, H3b) were partially supported: in contrast with Aghahabaei and colleagues’ study [15], the observed absence of significant associations was in line with Aghahabaei and Błachnio’s study [18]. On the other hand, from the theoretical perspective of the Dark Triad construct, both traits could be considered similar, as they share the common darker core of personality, i.e., callousness and disagreeableness [41], thus showing maladaptive tendencies and various adverse health outcomes, even if they are distinct in adopting cognitive strategies. Indeed, as expected, the positive relations of psychopathy with pleasure and engagement were supported (H2b), given that individuals high on psychopathy and characterized by a dysfunctional impulsivity seem to be more emotionally orientated and more focused on their flow experience, perceived as highly and intrinsically enjoyable, to seek short-term rewards. Consistently with the third hypothesis concerning psychopathy, it was found to be negatively associated with meaning (H2c), thus giving support to the previous negative associations with adverse health outcomes [33,34,43].

Like psychopaths, individuals high on Machiavellianism seem to adopt a hedonic path to obtain a positive reputation by others, thus confirming our expectation of the positive link between this trait and life of pleasure (H3a). In addition, the expected lack of associations of Machiavellianism with engagement and meaning was confirmed (H3c), in line with the features of Machs. In order to pursuit long-term goals, they tend to be more strategic-orientated and not to be totally engaged in their activities, because they calculate risk and benefits and, therefore, avoid feelings of euphoria that cannot be monitored. 

Generally, these first results supported the assumption that there are individual differences between Machiavellianism and psychopathy in adopting cognitive mechanisms related to the hedonic aspect of happiness. In parallel to the distinction based on the temporal function of life strategies, i.e., fast vs. slow, within the theoretical framework of life history theory [43], one can speculate on such distinction taking into account the role played by engagement. Obviously, future research is needed to replicate such associations in order to better clarify their nature.

Regarding narcissism, at first glance, the findings from correlations suggest that the trait was positively associated with the three paths and the two components of SWB measures. However, when looking at the findings from the path models, findings partially confirmed our three hypotheses. Contrary to our expectation (H1a), the indirect path from narcissism to SWB measures via the route of pleasure did not seem to play a significant effect, since no predictive power of pleasure emerged consistently with findings reporting stronger relationships for engagement and meaning than for pleasure [9,44]. Although narcissists tended to experience immediate pleasure, such a tendency did not foster frequent positive emotions. As for the supposed indirect links between narcissism and SWB via engagement (H1b), findings partially confirmed these relationships. Engagement played no mediating role in the relationship between this trait and life satisfaction, as expected narcissists tended to be more engagement-oriented in order to garner positive feedback and to experience higher pleasant emotions. In short, the ego reinforcement pursued by narcissists implies engaging activities that are likely socially oriented, following the agency model. It is this engagement, involving the agentic features of the trait, that highlights the bright side of narcissism. 

The third indirect effect on narcissism and satisfaction with life via life of meaning was not significant, in contrast with H1c. Even if narcissism was positively associated with meaning, individuals high on this trait sought personal harmony, leaving out activities related to the general good, such as community services. Consequently, the facets of an agency-communion model, as well as the similarity between narcissism and extraversion, were not supported by our findings.

Our study suffered from a number of limitations: (1) self-selected convenience sampling strategy encountered the problem of the data generalization; (2) self-report methodology based on self-administered questionnaires was subjected to respondents’ biases; (3) the face-valid measure to assess the Dark Triad traits and orientations to happiness was lacking; (4) the cross-sectional nature of our data could not determine the causality of the relationships; (5) the further components of Seligman’s revised theory were not considered; (6) other Cluster B personality traits, such as borderline personality trait, that involve dark traits (e.g., splitting of people) [45], repeated suicide [46] and impairment of cortical function, resulting in impulsivity [47], were not explored in detail in this study.

Nevertheless, this research provided an additional insight into the well-being literature, focusing on the connections between dark personalities and subjective well-being by examining the specific contribution of the three pathways to happiness. In conclusion, our results could suggest that the darker traits do not tend to seek happiness and SWB, whereas the bright side of narcissism highlights the pursuit of the emotional component of SWB by adopting engaging activities. Future research could further explore such connections by replicating the findings with more extensive measures and longitudinal designs that could offer a more precise determination of mediational processes.

## Figures and Tables

**Table 1:** Zero-order and partial correlations among the variables of interest.

	Pleasure	Meaning	Engagement	Life Satisfaction	Positive Affect	Negative Affect
Machiavellianism	0.313 *** 0.159 **	−0.044 −0.021	0.191 *** 0.053	−0.044 −0.032	0.090 0.012	0.202 *** 0.144 **
Narcissism	0.273 *** 0.143 **	0.180 *** 0.216 ***	0.231 *** 0.124 **	0.115 * 0.138 **	0.225 *** 0.173 ***	−0.095 * −0.173 ***
Psychopathy	0.377 ** 0.178 ***	−0.034 −0.100 *	0.228 ** 0.160 **	−0.023 −0.055	0.164 ** 0.059	0.143 ** 0.111 *

* *p* < 0.05; ** *p* < 0.010; *** *p* < 0.000. Zero-order correlations (r) are shown in the first row; partial correlations (sr) of each dark trait controlled for the other two dark traits are shown in the second row.

**Table 2:** Significant indirect effects of narcissism on life satisfaction and positive emotion.

Indirect Effect	95% CI	Point Estimate
Narcissism-> meaning -> life satisfaction	[−0.008, 0.146]	0.069
Narcissism-> engagement -> life satisfaction	[−0.008, 0.306]	0.149
Narcissism->engagement -> positive emotion	[0.016, 0.378]	0.197

Note. CI = 95% bias-corrected bootstrap confidence intervals.
